# Relation of the non-high-density lipoprotein cholesterol to high-density lipoprotein cholesterol ratio to residual risk in anticoagulated patients with atrial fibrillation: a report from the prospective Murcia AF Project III cohort

**DOI:** 10.1186/s12933-025-02927-x

**Published:** 2025-09-30

**Authors:** Eva Soler-Espejo, Yang Chen, José Miguel Rivera-Caravaca, María Pilar Ramos-Bratos, María Asunción Esteve-Pastor, Francisco Marín, Vanessa Roldán, Gregory Y. H. Lip

**Affiliations:** 1 Department of Hematology, Hospital Clínico Universitario Virgen de la Arrixaca, University of Murcia, Instituto Murciano de Investigación Biosanitaria Pascual Parrilla (IMIB-Pascual Parrilla), Murcia, Spain; 2 Liverpool Centre of Cardiovascular Science at University of Liverpool, Liverpool John Moores University and Liverpool Heart and Chest Hospital, William Henry Duncan Building, 6 West Derby Street, Liverpool, L7 8TX UK; 3 Faculty of Nursing, University of Murcia, Instituto Murciano de Investigación Biosanitaria Pascual Parrilla (IMIB-Pascual Parrilla), CIBERCV, Murcia, Spain; 4 Department of Cardiology, Hospital Clínico Universitario Virgen de la Arrixaca, University of Murcia, Instituto Murciano de Investigación Biosanitaria Pascual Parrilla (IMIB-Pascual Parrilla), CIBERCV, Murcia, Spain; 5 Department of Clinical Medicine, Aalborg University, Aalborg, Denmark; 6 Medical University of Bialystok, Bialystok, Poland

**Keywords:** Atrial fibrillation, Non-high-density lipoprotein cholesterol to high-density lipoprotein cholesterol ratio, Residual risk, Anticoagulation, Thromboembolism, MACE, Death

## Abstract

**Background:**

Atrial fibrillation (AF) confers a high risk of thromboembolism and cardiovascular events, which persists despite optimal oral anticoagulation (OAC). The non-high-density lipoprotein cholesterol (non-HDL-C) to high-density lipoprotein cholesterol (HDL-C) ratio (NHHR) integrates pro- and anti-atherogenic lipid fractions and has been linked to adverse outcomes in high-risk patient populations. The prognostic value of NHHR in anticoagulated AF patients is uncertain. We aimed to evaluate NHHR as a marker of residual thromboembolic and cardiovascular risk in this population.

**Methods:**

Consecutive AF outpatients initiating OAC between January 2016 and November 2021 were enrolled in this prospective cohort study. NHHR was calculated from baseline non-HDL-C and HDL-C levels, and patients were stratified into two groups. *Primary outcomes* were thromboembolic events (i.e., composite of ischaemic stroke (IS), transient ischaemic attack (TIA), or systemic embolism) and major adverse cardiovascular events (MACE), comprising myocardial infarction, IS, TIA, or cardiovascular death. *Secondary outcomes* included cardiovascular and all-cause death. Restricted cubic spline (RCS) models assessed non-linear associations, and multivariable Cox models evaluated associations between NHHR and outcomes.

**Results:**

1694 patients (52.8% female; age 76 years [IQR 69–82]; follow-up 1.86 years [SD 0.4]) were included. During follow-up, 97 (5.7%) experienced a thromboembolic event and 126 (7.4%) experienced MACE. RCS analysis showed significant linear associations between continuous NHHR and primary outcomes (p-overall < 0.001). High NHHR was independently associated with increased risk of thromboembolic events (adjusted Hazard Ratio [aHR] 2.15; 95% CI 1.41–3.29; *p* < 0.001) and MACE (aHR 1.69; 95% CI 1.15–2.48; *p* = 0.007), compared to the low NHHR group. No significant associations were observed for secondary outcomes.

**Conclusions:**

In anticoagulated AF patients, high NHHR was independently associated with increased residual thromboembolic and cardiovascular risk. NHHR may improve cardiovascular risk stratification in patients with AF, although external validation in more ethnically diverse cohorts is warranted.

**Graphical abstract:**

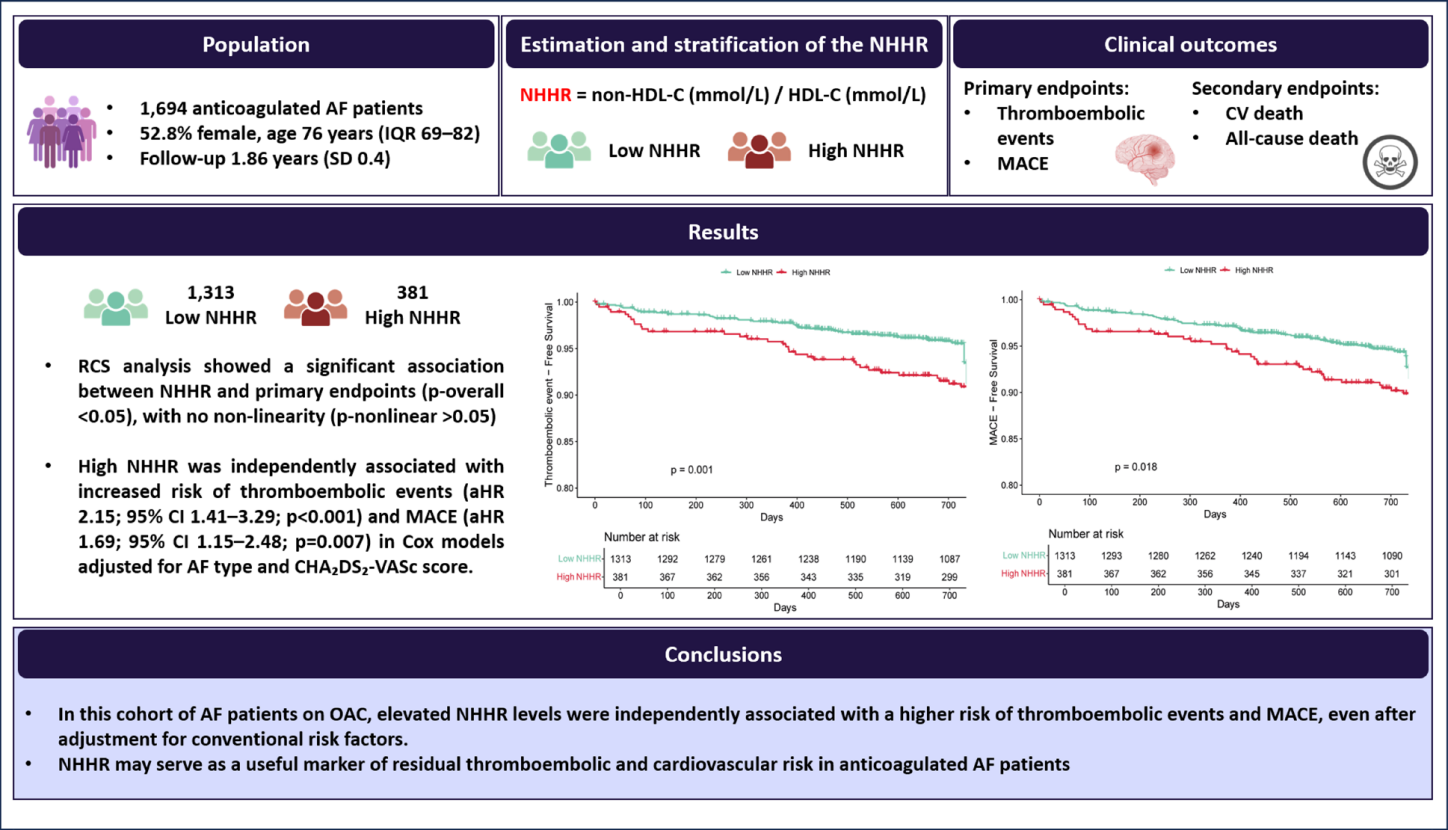

**Supplementary Information:**

The online version contains supplementary material available at 10.1186/s12933-025-02927-x.

## Background

Atrial fibrillation (AF) is the most common sustained cardiac arrhythmia and is associated with a substantially increased risk of thromboembolic events, particularly ischaemic stroke, as well as other major cardiovascular complications [1]. The widespread implementation of oral anticoagulation (OAC) therapy has led to significant reductions in thromboembolic risk [2]. Nevertheless, a notable residual risk persists even among patients who are appropriately anticoagulated [3], highlighting the need for additional biomarkers to enhance risk stratification beyond traditional scores [4, 5].

Dyslipidaemia is a well-established contributor to atherothrombotic disease [6]. Although low-density lipoprotein cholesterol (LDL-C) has historically been the primary lipid focus, increasing evidence supports the use of integrative lipid indices that more accurately reflect the balance between atherogenic and anti-atherogenic lipoproteins [7–9]. In this regard, the ratio of non-high-density lipoprotein cholesterol (non-HDL-C) to high-density lipoprotein cholesterol (HDL-C), termed the non-HDL-C to HDL-C ratio (NHHR), has emerged as a promising composite marker of net atherogenic burden [10].

Elevated NHHR has been linked to adverse cardiovascular outcomes in various populations, including individuals with coronary artery disease [11], pre-diabetes or diabetes [12, 13], hypertension [14], and chronic kidney disease [15]. A positive correlation with an increased prevalence of stroke has also been reported [16]. However, the prognostic value of NHHR in anticoagulated patients with AF remains largely unexplored, especially given the high residual risk of clinical events despite anticoagulation in these patients [17]. Given the multifactorial pathophysiology of thrombogenesis in AF, including contributions from endothelial dysfunction, inflammation, and hypercoagulability [18], there is strong biological plausibility for a lipid-derived marker such as NHHR to reflect residual thromboembolic and cardiovascular risk, even in the context of OAC therapy.

Accordingly, this study aimed to assess the association between NHHR levels and adverse cardiovascular outcomes in a prospective cohort of AF patients under OAC therapy.

## Methods

This prospective, observational cohort study, the Murcia AF Project III (MAFP-III), enrolled outpatients newly diagnosed with any type of AF who were naïve to oral anticoagulant (OAC) therapy and initiated treatment with either vitamin K antagonists (VKAs) or direct-acting oral anticoagulants (DOACs) at two anticoagulation clinics located in Murcia, Spain, between January 1, 2016, and November 30, 2021. Eligible participants were adults aged ≥ 18 years. Patients with prosthetic heart valves, rheumatic mitral valve disease, or other significant valvular abnormalities were excluded. No additional exclusion criteria were applied. All patients meeting the inclusion criteria were consecutively enrolled, as this was an all-comers study with no prior sample size calculation.

At baseline, comprehensive medical histories were obtained, including sociodemographic data, anthropometric measurements, comorbidities, and concomitant therapies. Stroke risk was assessed using the CHA_2_DS_2_-VASc and CHA_2_DS_2_-VA scores [19–21], while bleeding risk was evaluated using the HAS-BLED score [22].

The study protocol was approved by the Ethics Committees of the University Hospital Morales Meseguer (reference: EST:20/16) and the University Hospital Virgen de la Arrixaca (reference: 2020-11-12-HCUVA). It was conducted in accordance with the ethical principles of the 1964 Declaration of Helsinki and its subsequent amendments. Written informed consent was obtained from all participants prior to inclusion.

### Estimation and stratification of the NHHR

At baseline, laboratory assessments were conducted to evaluate the lipid profile, including measurements of serum total cholesterol and HDL-C levels. Following established methods [23], non-HDL-C was calculated using the formula: non-HDL (mmol/L) = total cholesterol (mmol/L) − HDL-C (mmol/L). The NHHR was then computed as [12, 24]: NHHR = non-HDL-C (mmol/L)/HDL-C (mmol/L). Participants were stratified into low and high NHHR groups based on a cutoff value identified through statistical analysis.

### Follow-up and clinical outcomes

Follow-up was conducted through in-person interviews during routine outpatient visits to the anticoagulation clinic, in alignment with standard clinical care. For patients who missed scheduled appointments, relevant information and vital status were obtained via review of medical records and follow-up telephone calls. As an observational study, all follow-up procedures were embedded within routine clinical practice, with no additional interventions or study-specific visits implemented.

The *primary endpoints* were: (i) the occurrence of thromboembolic events, defined as a composite of IS, transient ischaemic attack (TIA), or systemic embolism; and (ii) major adverse cardiovascular events (MACE), comprising myocardial infarction, IS, TIA, or cardiovascular death. *Secondary endpoints* included cardiovascular and all-cause death. Each adverse event was documented individually, and only the first occurrence of each event type was recorded. The follow-up period continued until the patient’s death or a maximum of 2 years, whichever occurred first. No patients were lost to follow-up.

### Statistical analysis

Continuous variables were expressed as mean ± standard deviation (SD) or median and interquartile range (IQR), as appropriate. Categorical variables were presented as absolute frequencies and percentages. Group comparisons were performed using Student’s *t*-test or the Mann–Whitney U test for continuous variables, and Fisher’s exact test or Pearson’s chi-square test for categorical variables.

The cut-off value for NHHR grouping was determined using the ‘survminer’ package in R, based on maximally selected rank statistics to optimize separation of time-to-event outcomes. To assess both overall and non-linear associations between NHHR and the primary outcomes, restricted cubic spline (RCS) analyses were conducted with NHHR as a continuous variable. The reference point for the splines was set at the identified cut-off. The number of knots was determined based on the Akaike Information Criterion (AIC) and Bayesian Information Criterion (BIC), with the final models employing three knots, placed at the 10th, 50th, and 90th percentiles of the index distribution.

Incidence rates with 95% Poisson confidence intervals (CIs) for the primary outcome were calculated for each NHHR group. Rate differences (RDs) were estimated using the low NHHR group as the reference.

Kaplan–Meier survival curves were constructed to depict event-free survival across NHHR groups, with comparisons made using the log-rank test.

Then, multivariable Cox proportional hazards models were employed to assess the independent association between NHHR and both primary and secondary outcomes, treating NHHR as a categorical variable. The following models were applied: Model 1: unadjusted; Model 2: adjusted for AF type and CHA_2_DS_2_-VASc score. These covariates were selected a priori because they are well-established determinants of thromboembolic and cardiovascular risk in AF, consistent with previous studies [25]. Adjusted hazard ratios (aHRs) with corresponding 95% CIs were reported. The proportional hazards assumption was tested using Schoenfeld residuals, and no significant violations were observed. Additionally, a sensitivity analysis was performed by including smoking habit, diuretics, antilipemic agents, and antiplatelet therapy as separate covariates in the multivariable models, alongside AF type and CHA_2_DS_2_-VASc score, to further assess the robustness of the observed associations.

Subgroup analyses were conducted to evaluate the consistency of associations between NHHR and both thromboembolic events and MACE across clinically relevant strata (including age, sex, AF type, CHA_2_DS_2_-VASc score, hypertension, diabetes, history of stroke/TIA, kidney disease, and antilipemic therapy). These analyses were based on formal tests of interaction by including multiplicative interaction terms in the fully adjusted model. Since we did not conduct or interpret multiple within-group effect estimates separately, but rather focused on the statistical significance of interaction terms to assess effect modification, correction for multiple comparisons was not applied.

All statistical tests were two-sided, and a *p*-value < 0.05 was considered statistically significant. Analyses were performed using STATA v18.0 (StataCorp LLC, College Station, TX, USA), R (R Foundation for Statistical Computing, Vienna, Austria), and SPSS v25.0 (IBM Corp., Armonk, NY, USA).

## Results

The overall cohort comprised 3259 AF patients (52.8% female; median age 77 years, IQR 70–83). Among them, 1694 patients (52.8% female; median age 76 years, IQR 69–82) had complete lipid profile data, including total cholesterol and HDL-C, and were included in the present analysis. All were exclusively receiving DOACs. Patients with and without complete lipid data were comparable in baseline characteristics.

The median CHA_2_DS_2_-VASc score was 4 (IQR 3–5), the CHA_2_DS_2_-VA score was 4 (IQR 2–5), and the HAS-BLED score was 3 (IQR 2–4). Regarding lipid parameters, the median total cholesterol was 4.2 mmol/L (IQR 3.5–4.8), HDL-C was 1.3 mmol/L (IQR 1.0–1.6), non-HDL-C was 2.8 mmol/L (IQR 2.3–3.5), and the median NHHR was 2.2 (IQR 1.6–2.9). Baseline characteristics of the study cohort are summarised in Table 1.

**Table 1:** Baseline clinical characteristics of the study population

	N = 1694
*Demographics*	
Age, median (IQR)	76 [69–82]
Sex [Female], n (%)	895 (52.8)
AF type, n (%)	
Persistent	1022 (60.3)
Paroxysmal	672 (39.7)
*Comorbidities, n (%)*	
Hypertension	1449 (85.5)
Diabetes mellitus	647 (38.2)
Heart failure	301 (17.8)
History of stroke/TIA/thromboembolism	454 (26.8)
Vascular disease*	356 (21)
Renal impairment	387 (22.8)
Dyslipidaemia	989 (58.4)
COPD/OSA	374 (22.1)
History of relevant bleeding	266 (15.7)
Liver disease	61 (3.6)
History of cancer	222 (13.1)
Smoking habit	483 (28.5)
Alcoholism	180 (10.6)
*Concomitant treatment, n (%)*	
Antiarrhythmics	329 (19.4)
ACE inhibitors	429 (25.3)
ARBs	768 (45.3)
Calcium channel blockers	493 (29.1)
Beta-blockers	1114 (65.8)
Diuretics	1023 (60.4)
Antilipemic agents	988 (58.3)
Statins	183 (10.8)
Others (Ezetimibe, fenofibrates…)	389 (23)
Combined treatment	416 (24.6)
Oral hypoglycemic agents	534 (31.5)
Insulin	146 (8.6)
Antiplatelet therapy	168 (9.9)
*Analytical parameters, median (IQR)*	
Total colesterol (mmol/L)	4.2 [3.5–4.8]
HDL-C (mmol/L)	1.3 [1.0–1.6]
Non-HDL-C (mmol/L)	2.8 [2.3–3.5]
NHHR	2.2 [1.6–2.9]
*Stroke and bleeding scores, median (IQR)*	
CHA_2_DS_2_-VA	4 [2–5]
CHA_2_DS_2_-VASc	4 [3–5]
HAS-BLED	3 [2–4]

NHHR, non-high-density lipoprotein cholesterol to high-density lipoprotein cholesterol ratio; IQR, interquartile range; TIA, transient ischaemic attack; COPD/OSA, chronic obstructive pulmonary disease/obstructive sleep apnea; ACE inhibitors, angiotensin-converting-enzyme inhibitors; ARBs, angiotensin II receptors blockers; HDL-C, high-density lipoprotein cholesterol

*Vascular disease includes coronary artery disease and/or peripheral artery disease

**Fig. 1:**
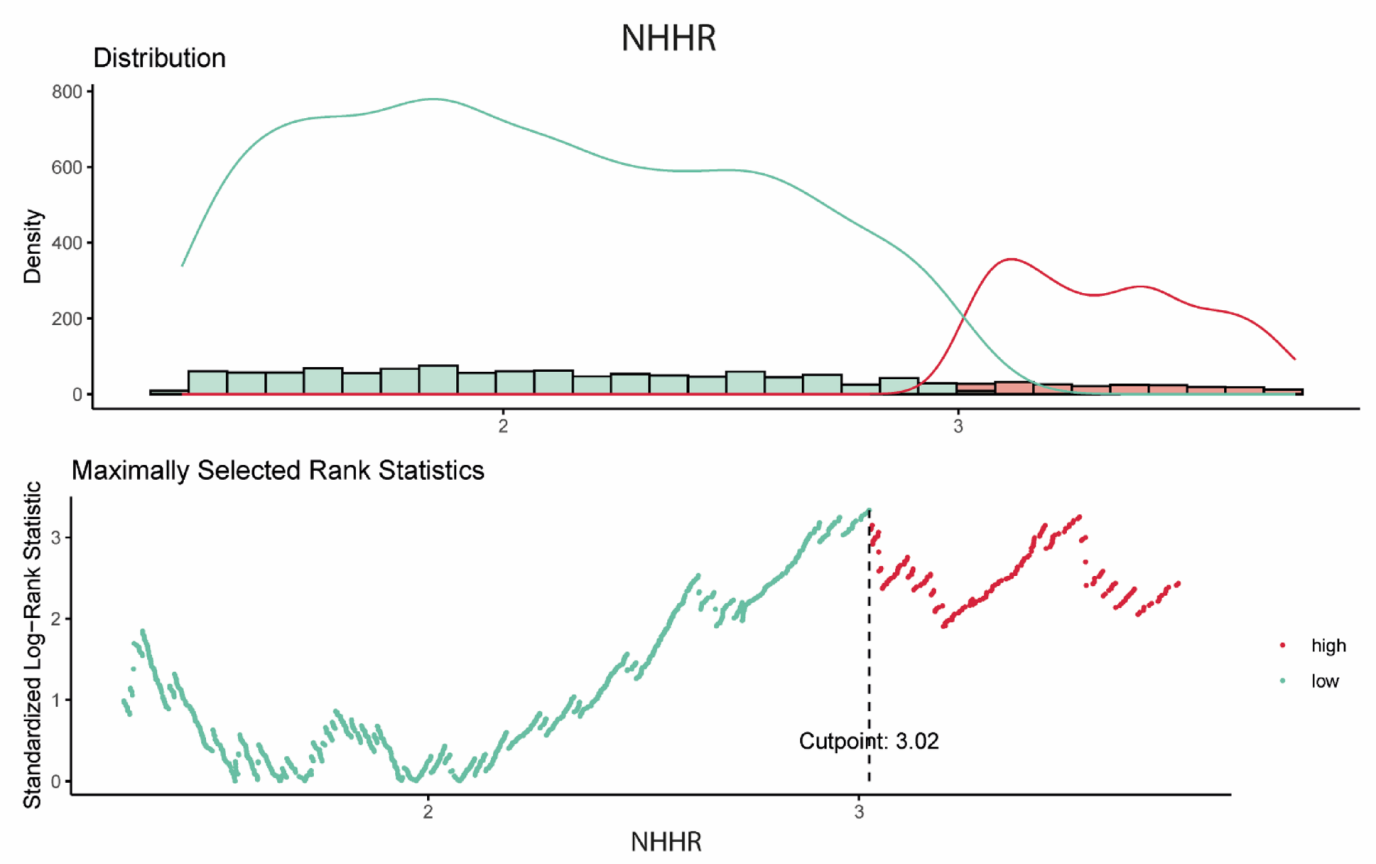
Visualisation of the optimal cut-off point for thromboembolic events. NHHR, non-high-density lipoprotein cholesterol to high-density lipoprotein cholesterol ratio

Based on the predefined NHHR cut-off (Fig. 1), 1313 patients (77.5%) were classified into the low NHHR group and 381 (22.5%) into the high NHHR group. Baseline characteristics according to NHHR category are presented in Supplementary Table 1. Patients in the low NHHR group were significantly older and more likely to be female compared to those in the high NHHR group (*p* < 0.001, for each). Conversely, the high NHHR group had a higher proportion of male patients and a significantly greater prevalence of smoking (*p* < 0.001, for each). No substantial differences in overall comorbidity burden were observed between the two groups.

### Primary outcomes across NHHR groups

During a mean follow-up of 1.86 years (SD 0.4 years), 97 patients (5.7%) experienced a thromboembolic event and 126 (7.4%) experienced a MACE. As shown in Table 2, incidence rates for both thromboembolic events (2.57 *vs*. 4.94 events per 100 person-years; *p* = 0.009) and MACE (3.58 *vs*. 5.49 events per 100 person-years; *p* = 0.048) were significantly lower in the low NHHR group compared to the high NHHR group.

**Table 2:** Incidence rate and rate difference for the different outcomes according to the NHHR group

	Low NHHR [Ref]		High NHHR		RD (95% CI)	*p *value
	N (%)	Incidence rate* (95% CI)	N (%)	Incidence rate* (95% CI)		
Any thromboembolic event	63 (4.8)	2.57 (1.98–3.29)	34 (8.9)	4.94 (3.42–6.90)	2.37 (0.59–4.15)	0.009
MACE	88 (6.7)	3.58 (2.87–4.40)	38 (10)	5.49 (3.88–7.53)	1.91 (0.01–3.81)	0.048
Cardiovascular death	41 (3.1)	1.64 (1.17–2.22)	12 (3.1)	1.66 (0.86–2.90)	0.02 (− 1.04 to 1.09)	0.964
All-cause death	128 (9.7)	5.11 (4.26–6.08)	34 (8.9)	4.71 (3.26–6.58)	− 0.40 (− 2.22 to 1.41)	0.663

NHHR, non-high-density lipoprotein cholesterol to high-density lipoprotein cholesterol ratio; CI, confidence interval; RD, rate difference; MACE, major adverse cardiovascular events

*Per 100 person-years

### Secondary outcomes across NHHR groups

During the follow-up period, 53 patients (3.1%) died from cardiovascular causes and 162 (9.6%) died from any cause. No statistically significant differences were observed between NHHR groups in terms of cardiovascular death (1.64 vs. 1.66 events per 100 person-years; *p* = 0.964) or all-cause death (5.11 vs. 4.71 events per 100 person-years; *p* = 0.361) (Table 2).

### Association of thromboembolic events with NHHR groups

**Fig. 2:**
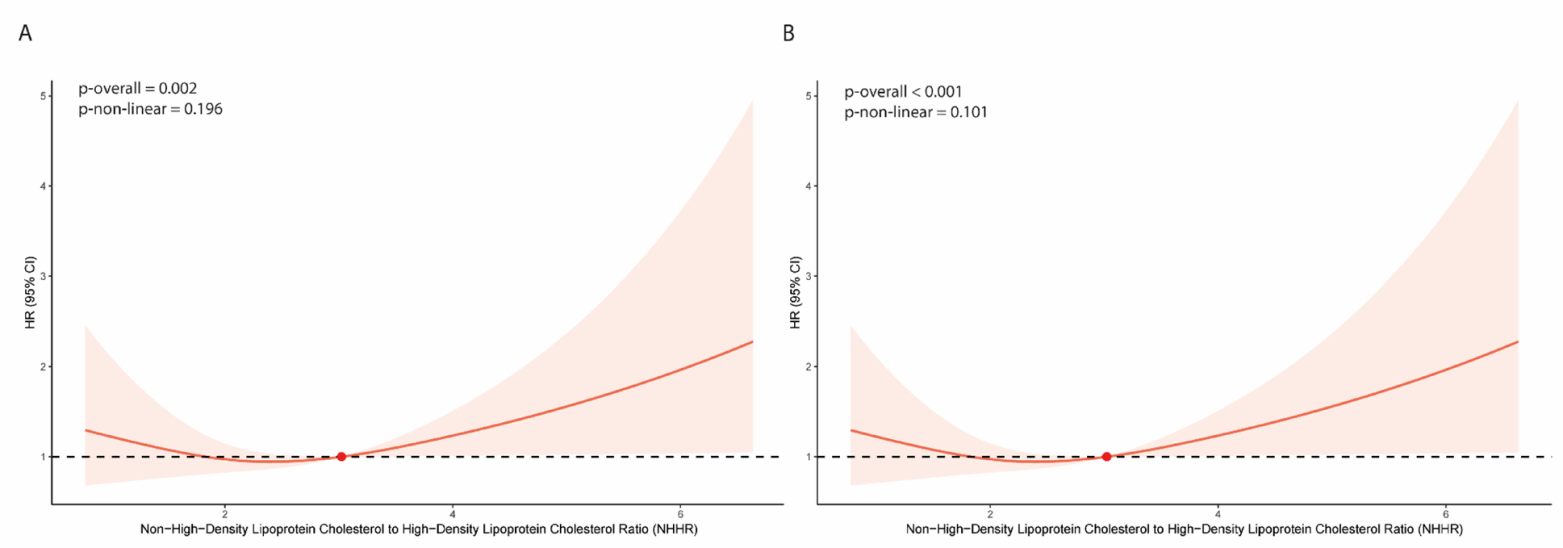
Restricted cubic spline analysis for thromboembolic events (**A**) and MACE (**B**). HR, hazard ratio; CI, confidence interval

**Fig. 3:**
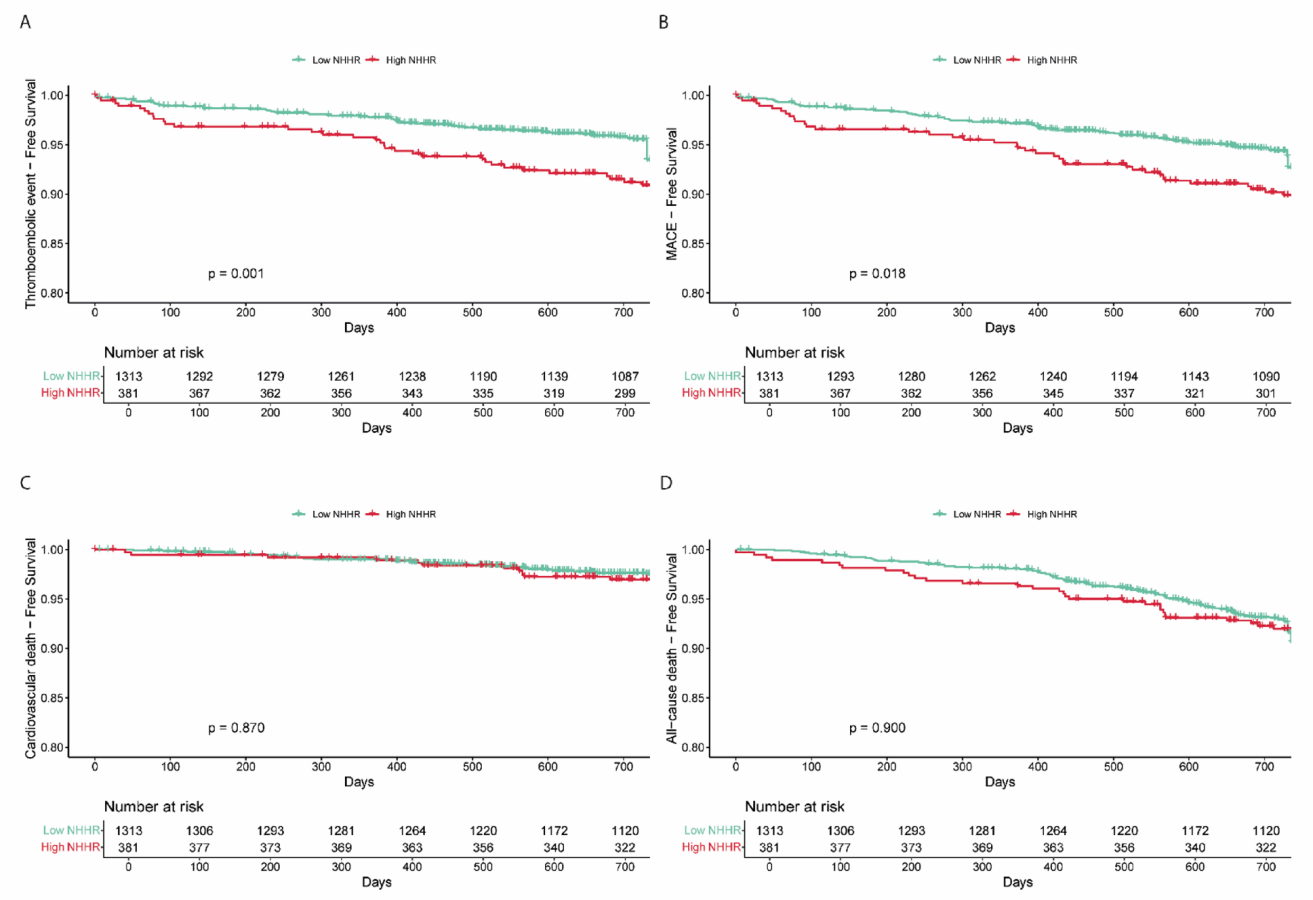
Kaplan Meier curves for the different outcomes in NHHR group. NHHR, non-high-density lipoprotein cholesterol to high-density lipoprotein cholesterol ratio

Restricted cubic spline (RCS) analysis demonstrated a significant overall association between NHHR levels and thromboembolic risk (p-overall = 0.002), with no evidence of non-linearity (p-non-linear = 0.196) (Fig. 2a). Kaplan–Meier analysis showed significantly lower event-free survival in the high NHHR group compared to the low NHHR group (log-rank test, *p* < 0.001) (Fig. 3).

As presented in Table 3, unadjusted Cox proportional hazards models indicated that patients in the high NHHR group had a significantly increased risk of thromboembolic events compared to those in the low NHHR group (HR 2.02; 95% CI 1.33–3.08; *p* = 0.001). This association remained statistically significant after adjustment for AF type and CHA_2_DS_2_-VASc score (aHR 2.15; 95% CI 1.41–3.29; *p* < 0.001), and was further confirmed in sensitivity analyses adjusted for smoking habit, diuretics, antilipemic agents, and antiplatelet therapy (Supplementary Table 2).

**Table 3:** Hazard ratio for the different outcomes according to the NHHR group

	N (%)	Model I		Model II	
		HR (95% CI)	*p* value	aHR (95% CI)	*p *value
Any thromboembolic event					
Low NHHR	63 (4.8)	Reference		Reference	
High NHHR	34 (8.9)	2.02 (1.33–3.08)	0.001	2.15 (1.41–3.29)	< 0.001
MACE					
Low NHHR	88 (6.7)	Reference		Reference	
High NHHR	38 (10)	1.58 (1.08–2.31)	0.019	1.69 (1.15–2.48)	0.007
Cardiovascular death					
Low NHHR	41 (3.1)	Reference		Reference	
High NHHR	12 (3.1)	1.06 (0.55–2.01)	0.870	1.12 (0.59–2.14)	0.732
All-cause death					
Low NHHR	128 (9.7)	Reference		Reference	
High NHHR	34 (8.9)	0.98 (0.67–1.43)	0.900	1.05 (0.72–1.53)	0.811

Model I: unadjusted

Model II: adjusted for AF type, and CHA_2_DS_2_-VASc score

NHHR, non-high-density lipoprotein cholesterol to high-density lipoprotein cholesterol ratio; aHR, adjusted hazard ratio; CI, confidence interval; MACE, major adverse cardiovascular events

### Association of MACE with NHHR groups

RCS analysis revealed a significant overall association between NHHR levels and the risk of MACE (p-overall < 0.001), without evidence of non-linearity (p-non-linear = 0.101) (Fig. 2b). Kaplan–Meier analysis demonstrated significantly lower event-free survival in the high NHHR group compared to the low NHHR group (log-rank *p* = 0.018) (Fig. 3).

As shown in Table 3, unadjusted Cox proportional hazards models indicated that patients in the high NHHR group had an increased risk of MACE compared to those in the low NHHR group (HR 1.58; 95% CI 1.08–2.31; *p* = 0.019). This association remained statistically significant after adjustment for AF type and CHA_2_DS_2_-VASc score (aHR 1.69; 95% CI 1.15–2.48; *p* = 0.007), and the findings were supported in sensitivity analyses additionally adjusted for smoking habit, diuretics, antilipemic agents, and antiplatelet therapy (Supplementary Table 2).

### NHHR groups and secondary outcomes

Kaplan–Meier curves showed no significant differences between NHHR groups in terms of cardiovascular death (log-rank *p* = 0.870) or all-cause death (log-rank *p* = 0.900) (Fig. 3). Similarly, Cox proportional hazards models adjusted for AF type and CHA_2_DS_2_-VASc score did not indicate a statistically significant increased risk in the high NHHR group compared to the low NHHR group for cardiovascular death (aHR 1.12; 95% CI 0.59–2.14; *p* = 0.732) or all-cause death (aHR 1.05; 95% CI 0.72–1.53; *p* = 0.811). These null associations remained unchanged in sensitivity analyses (Supplementary Table 2).

### Subgroup analysis

The association between elevated NHHR levels and the risk of thromboembolic events and MACE remained directionally consistent across all clinical subgroups, with no significant interactions observed (all p-for-interaction > 0.05).

**Fig. 4:**
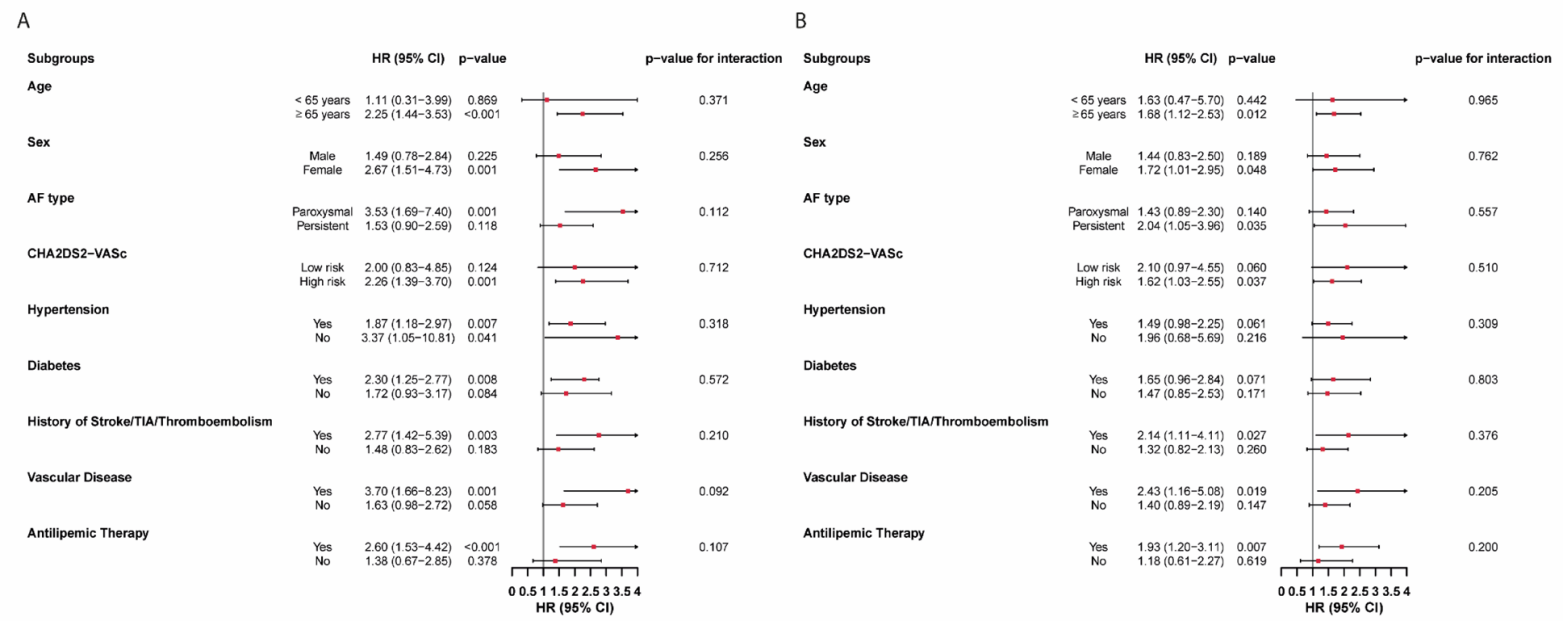
Hazard ratios for NHHR across clinical subgroups for thromboembolic events (**A**) and MACE (**B**), derived from Cox regression analysis. AF, atrial fibrillation; TIA, transient ischaemic attack; HR, hazard ratio; CI, confidence interval

Statistically significant associations with thromboembolic risk were observed in specific subgroups, including patients aged ≥ 65 years (*p* =  < 0.001), female patients (*p* = 0.001), those with paroxysmal AF (*p* = 0.001), a high CHA_2_DS_2_-VASc score (*p* = 0.001), hypertension (*p* = 0.007), no hypertension (*p* = 0.041), diabetes (*p* = 0.008), a history of stroke/TIA/thromboembolism (*p* = 0.003), vascular disease (*p* = 0.001) and those with antilipemic therapy (*p* =  < 0.001) (Fig. 4a).

Similarly, significant associations with MACE were found in subgroups of patients aged ≥ 65 years (*p* = 0.012), female sex (*p* = 0.048), persistent AF (*p* = 0.035), and those with a history of stroke/TIA/thromboembolism (*p* = 0.027), vascular disease (*p* = 0.019) and those with antilipemic therapy (*p* = 0.007) (Fig. 4b).

## Discussion

In this large, prospective cohort of anticoagulated AF patients, our principal findings are as follows: (i) patients with elevated NHHR values had nearly twice the incidence of thromboembolic events and a significantly higher rate of MACE compared to those with lower NHHR values; (ii) high NHHR was independently associated with an increased risk of both thromboembolic events and MACE, even after adjustment for conventional risk factors; (iii) RCS analysis confirmed a significant, linear, dose–response relationship between NHHR and both adverse outcomes; (iv) event-free survival was significantly lower in the high NHHR group for both thromboembolic events and MACE; and (v) the association between elevated NHHR and the risk of adverse outcomes remained consistent across all clinically relevant subgroups.

To our knowledge, this is the first prospective study to investigate the prognostic relevance of NHHR in characterising residual cardiovascular risk among anticoagulated AF patients. This population represents a distinct clinical phenotype [26], defined by a complex interplay of prothrombotic mechanisms and metabolic dysregulation, in which lipid-related biomarkers may offer additional risk stratification beyond conventional scoring systems [27]. Although previous studies have examined associations between elevated NHHR and cardiovascular outcomes, these have primarily involved general populations [16] or individuals with highly atherogenic comorbidities, such as coronary artery disease [11], pre-diabetes or diabetes [12, 13], hypertension [14], with none to date specifically addressing its prognostic value in AF patients.

Despite adequate OAC, a substantial proportion of AF patients continue to experience thromboembolic events [5, 28], highlighting the limitations of current clinical scores and underscoring the need to uncover non-thrombin-related mechanisms contributing to residual risk. From a pathophysiological standpoint, the association between elevated NHHR and adverse cardiovascular outcomes in anticoagulated AF patients is biologically plausible and supported by several mechanistic pathways [29]. AF is not merely an arrhythmia, but a systemic disorder characterised by a prothrombotic state involving endothelial dysfunction, blood stasis, and hypercoagulability [30]. Within this milieu, dyslipidaemia contributes to thrombogenesis via multiple interrelated mechanisms. Elevated levels of atherogenic lipoproteins, particularly non-HDL particles such as VLDL and LDL, promote oxidative stress, endothelial injury, and inflammatory activation [31], thereby enhancing platelet adhesion and coagulation cascade activation. Conversely, HDL-C exerts antithrombotic, anti-inflammatory, and antioxidative effects by preserving endothelial function and inhibiting LDL oxidation and monocyte adhesion [32, 33]. An increased NHHR thus reflects an imbalance in the lipoprotein profile that favours a prothrombotic and pro-inflammatory state.

Furthermore, non-HDL-C has been independently associated with elevated levels of tissue factor and thrombin-antithrombin complexes [34], while reduced HDL-C correlates with impaired fibrinolysis and diminished nitric oxide bioavailability [35]. These mechanisms are particularly relevant in AF patients, where atrial structural remodelling, systemic inflammation, and metabolic dysregulation converge to amplify thrombotic risk [29, 30]. Consequently, NHHR may serve as a surrogate of lipid-driven vascular dysfunction and thromboinflammation, capturing dimensions of residual risk that remain unaccounted for in traditional clinical models, even in the presence of effective OAC therapy.

Despite the absence of direct comparisons, our findings are in line with existing literature. For instance, Ma et al. [16] examined the relationship between NHHR levels and the incidence of stroke in 29,928 participants from the NHANES study. Patients were divided into quartiles based on NHHR levels, and compared to the first quartile, there was a significant increase in the risk of stroke in the higher quartiles (odds ratio [OR] 1.35; 95% CI 1.08–1.69; OR 1.83; 95% CI 1.42–2.36; and OR 2.04; 95% CI 1.50–2.79, for each successive quartile, respectively).

On the other hand, Wang et al. [36] examined the relationship between NHHR levels and the incidence of cardiovascular disease (CVD) in 4629 participants aged ≥ 45 years from the CHARLS cohort. Compared to those in the lowest NHHR tertile, individuals in the highest tertile for both baseline and cumulative NHHR exhibited a significantly increased risk of CVD (aHR 1.43; 95% CI 1.21–1.70; and aHR 1.45; 95% CI 1.23–1.72, respectively).

While clinical scores such as CHA_2_DS_2_-VASc or CHA_2_DS_2_-VA remain the cornerstone of thromboembolic risk stratification in AF, their predictive performance is modest [37]. These tools primarily account for clinical comorbidities and neglect key metabolic and inflammatory contributors to thrombogenesis. In this context, NHHR may provide incremental prognostic value by integrating both atherogenic burden and the anti-inflammatory, antithrombotic properties of HDL-C. Unlike isolated lipid parameters (e.g., LDL-C or HDL-C), NHHR reflects a functional lipid imbalance that may be especially relevant in the prothrombotic milieu characteristic of AF. As such, it could serve as a useful complement to conventional risk scores, particularly in patients with borderline risk or discordant clinical features. Although mathematically related to the TC/HDL-C ratio (NHHR = [TC/HDL-C] − 1), we deliberately employed NHHR to ensure methodological consistency with prior studies [12, 24] and because non-HDL-C is an established, guideline-endorsed therapeutic target. Expressing the ratio in this way thus confers greater clinical and pathophysiological relevance, as it more directly integrates the balance between atherogenic and protective lipoprotein fractions.

Our subgroup analyses further underscore the robustness and clinical relevance of NHHR as a prognostic marker. The associations between elevated NHHR and both thromboembolic events and MACE remained directionally consistent across all predefined strata, with no significant effect modification. Importantly, significant associations were observed in subgroups traditionally considered at heightened cardiovascular risk, including older adults (≥ 65 years), women, and individuals with prior thromboembolic events or vascular disease.

Notably, the sex-specific findings warrant further consideration. The stronger association observed in female patients may reflect recognized differences in lipid metabolism, endothelial function, and thromboembolic risk in AF [38]. While recent European studies [39, 40] have suggested that sex disparities in stroke risk may be diminishing in contemporary cohorts, such differences remain evident in certain populations [21]. Our results suggest that NHHR may capture residual cardiovascular risk more effectively in women, supporting the hypothesis that sex-specific vascular and metabolic profiles influence prognostic markers in AF. These findings highlight the need for further investigation into tailored risk stratification strategies that account for sex-based biological variation.

Given its simplicity, availability, and low cost, NHHR could be readily incorporated into routine clinical practice. Our findings suggest that it may help reclassify residual cardiovascular risk in anticoagulated AF patients, especially those who remain at elevated risk despite appropriate OAC therapy. NHHR could support more intensive surveillance, guide adjunctive therapeutic strategies, or inform personalised lifestyle interventions aimed at improving lipid profiles. Importantly, because NHHR integrates both detrimental and protective lipid components, it may function as a dynamic biomarker for longitudinal risk monitoring and could be responsive to pharmacological or lifestyle modifications.

Looking ahead, several avenues for research should be pursued. External validation in independent and ethnically diverse cohorts is required to confirm the robustness and generalisability of these findings. Mechanistic studies could further elucidate the biological pathways linking NHHR to thromboinflammation and atrial remodelling. Prospective interventional trials are warranted to assess whether NHHR-guided risk stratification, lipid-lowering therapies (e.g., statins, fibrates, or targeted nutritional approaches), and comprehensive lifestyle interventions [41] (e.g., diet, physical activity, and weight management) can effectively mitigate residual cardiovascular risk. In addition, as direct biochemical quantification of lipoprotein fractions becomes more accessible, future studies should determine whether such methods offer incremental precision over calculated NHHR, particularly in patients with borderline lipid values. Finally, evaluating longitudinal changes in NHHR in relation to evolving risk or treatment response would further reinforce its role as a clinically actionable biomarker.

### Limitations

This study has several limitations that warrant consideration. First, due to its observational design, causal inferences cannot be definitively established. Although multivariable adjustment was performed for a comprehensive set of confounders, residual confounding from unmeasured or unknown variables cannot be entirely ruled out. Second, all participants were anticoagulated at baseline, which limits the applicability of our findings to anticoagulant-naïve individuals or those managed with alternative antithrombotic strategies or non-pharmacological approaches. Third, the cohort was predominantly Caucasian and recruited from two academic centres in Spain. This demographic and geographic homogeneity may limit the generalisability of the findings to more ethnically diverse or globally representative populations, particularly given known racial and ethnic differences in AF epidemiology, and AF-related complications such as stroke and bleeding [42, 43]. Fourth, lipid parameters, including total cholesterol and HDL-C, were measured only at baseline. Although this is a common approach in biomarker research, it does not capture intra-individual variability over time or the impact of changes due to comorbidities, lifestyle interventions, or lipid-lowering therapies. Emerging evidence suggests that longitudinal changes in lipid markers may have stronger prognostic implications for MACE than single measurements [44]. Fifth, NHHR was calculated indirectly using the non-HDL-C-to-HDL-C ratio rather than direct biochemical quantification. While this approach is well validated and clinically accepted, it may be subject to minor inaccuracies, particularly in individuals with borderline lipid values. Sixth, the incidence of certain outcomes, especially cardiovascular death, was relatively low in specific NHHR strata, which may have reduced statistical power and led to wider confidence intervals for some effect estimates. Lastly, we did not assess other lipid-derived or inflammatory biomarkers (e.g., apolipoprotein B, lipoprotein(a), or high-sensitivity C-reactive protein), which may interact with or complement NHHR in predicting cardiovascular risk.

## Conclusion

In this cohort of AF patients on OAC, elevated NHHR levels were independently associated with a higher risk of thromboembolic events and MACE, even after adjustment for conventional risk factors. NHHR may serve as a useful marker of residual thromboembolic and cardiovascular risk in anticoagulated AF patients. However, external validation in more ethnically diverse and geographically representative cohorts is warranted to confirm the generalizability of these findings.

## Supplementary Information

Below is the link to the electronic supplementary material.

Supplementary Material 1

## Data Availability

Derived data supporting the findings of this study are available from the corresponding author, Yang Chen, on reasonable request.
