# Supplementary material for: Relation of the non-high-density lipoprotein cholesterol to high-density lipoprotein cholesterol ratio to residual risk in anticoagulated patients with atrial fibrillation: a report from the prospective Murcia AF Project III cohort
Source: Cardiovasc Diabetol. 2025 Sep 30;24:374. doi: 10.1186/s12933-025-02927-x (PMC12486827; doi:10.1186/s12933-025-02927-x)
Supplement: Supplementary file 1 — Supplementary Material 1 [file 12933_2025_2927_MOESM1_ESM.docx]

| **Supplementary Table 1. Baseline clinical characteristics according to NHHR group.** | | | |  |  |
| --- | --- | --- | --- | --- | --- |
|  | **Low NHHR**  **(N= 1,313)** | **High NHHR**  **(N=381)** | **p-value** | |  |
| **Demographics** |  |  |  | |  |
| Age, median (IQR) | 77 [70-83] | 73 [65-81] | <0.001 | |  |
| Sex [Female], n (%) | 727 (55.4) | 168 (44.1) | <0.001 | |  |
| AF type, n (%) |  |  |  | |  |
| Persistent | 794 (60.5) | 228 (59.8) | 0.872 | |  |
| Paroxysmal | 519 (39.5) | 153 (40.2) |  | |  |
|  |  |  |  | |  |
| **Comorbidities, n (%)** |  |  |  | |  |
| Hypertension | 1,124 (85.6) | 325 (85.3) | 0.948 | |  |
| Diabetes mellitus | 488 (37.2) | 159 (41.7) | 0.120 | |  |
| Heart failure | 237 (18.1) | 64 (16.8) | 0.626 | |  |
| History of stroke/TIA/thromboembolism | 344 (26.2) | 110 (28.9) | 0.332 | |  |
| Vascular disease* | 290 (22.1) | 66 (17.3) | 0.053 | |  |
| Renal impairment | 302 (23) | 35 (22.3) | 0.831 | |  |
| Dyslipidaemia | 774 (58.9) | 215 (56.4) | 0.413 | |  |
| COPD/OSA | 281 (21.4) | 93 (24.4) | 0.240 | |  |
| History of relevant bleeding | 203 (15.5) | 63 (16.5) | 0.669 | |  |
| Liver disease | 49 (3.7) | 12 (3.1) | 0.703 | |  |
| History of cancer | 177 (13.5) | 45 (11.8) | 0.445 | |  |
| Smoking habit | 347 (26.4) | 136 (35.7) | <0.001 | |  |
| Alcoholism | 131(10) | 49 (12.9) | 0.130 | |  |
|  |  |  |  | |  |
| **Concomitant treatment, n (%)** | | | |  |  |
| Antiarrhythmics | 254 (19.3) | 75 (19.7) | 0.941 | |  |
| ACE inhibitors | 332 (25.3) | 97 (25.5) | 0.999 | |  |
| ARBs | 603 (45.9) | 165 (43.3) | 0.398 | |  |
| Calcium channel blockers | 385 (29.3) | 108 (28.3) | 0.760 | |  |
| Beta-blockers | 850 (64.7) | 264 (69.3) | 0.112 | |  |
| Diuretics | 811 (61.8) | 212 (55.6) | 0.036 | |  |
| Antilipemic agents | 789 (60.1) | 199 (52.2) | <0.001 | |  |
| Statins | 120 (15.2) | 63 (31.7) |  | |  |
| Others (Ezetimibe, fenofibrates...) | 339 (43.0) | 50 (25.1) |  | |  |
| Combined treatment | 330 (41.8) | 86 (43.2) |  | |  |
| Oral hypoglycemic agents | 399 (30.4) | 135 (35.4) | 0.071 | |  |
| Insulin | 113 (8.6) | 33 (8.7) | 0.999 | |  |
| Antiplatelet therapy | 138 (10.5) | 30 (7.9) | 0.156 | |  |
|  |  |  |  | |  |
| **Analytical parameters, median (IQR)** | | | |  |  |
| Total colesterol (mmol/L) | 4.0 [3.4-4.7] | 4.8 [4.2-5.5] | <0.001 | |  |
| HDL-C (mmol/L) | 1.2 [1.1-1.6] | 1 [0.8-1.1] | <0.001 | |  |
| Non-HDL-C (mmol/L) | 2.6 [2.1-3.1] | 3.8 [3.3-4.3] | <0.001 | |  |
| NHHR | 1.9 [1.5-2.4] | 3.6 [3.3-4.3] | <0.001 | |  |
|  |  |  |  | |  |
| **Stroke and bleeding scores, median [IQR]** | | | |  |  |
| CHA_2_DS_2_-VA | 4 [3-5] | 3 [2-5] | 0.016 | |  |
| CHA_2_DS_2_-VASc | 4 [3-5] | 4 [2-5] | <0.001 | |  |
| HAS-BLED | 3 [2-4] | 3 [2-4] | 0.012 | |  |
| Abbreviations: NHHR, non-high-density lipoprotein cholesterol to high-density lipoprotein cholesterol ratio; IQR, interquartile range; TIA, transient ischemic attack; COPD/OSA, chronic obstructive pulmonary disease/obstructive sleep apnea; ACE inhibitors, angiotensin-converting-enzyme inhibitors; ARBs, angiotensin II receptors blockers; HDL-C, high-density lipoprotein cholesterol.  *Vascular disease includes coronary artery disease and/or peripheral artery disease. | | | |  |  |

**Supplementary Table 2.** **Sensitivity analysis for the different outcomes according to the NHHR group.**

|  | **N (%)** | **aHR ***  **(95% CI)** | **p-value** |  | |
| --- | --- | --- | --- | --- | --- |
| **Any thromboembolic event** |  |  |  |  |  |
| Low NHHR | 63 (4.8) | Reference |  |  | |
| High NHHR | 34 (8.9) | 2.11 (1.38–3.23) | <0.001 |  | |
| **MACE** |  |  |  |  | |
| Low NHHR | 88 (6.7) | Reference |  |  | |
| High NHHR | 38 (10) | 1.62 (1.11–2.38) | 0.019 |  | |
| **Cardiovascular death** |  |  |  |  | |
| Low NHHR | 41 (3.1) | Reference |  |  | |
| High NHHR | 12 (3.1) | 1.08 (0.56–2.06) | 0.824 |  | |
| **All-cause death** |  |  |  |  | |
| Low NHHR | 128 (9.7) | Reference |  |  | |
| High NHHR | 34 (8.9) | 1.01 (0.69–1.48) | 0.959 |  | |
| * adjusted for smoking habit, diuretics, antilipemic agents, and antiplatelet therapy.  Abbreviations: NHHR, non-high-density lipoprotein cholesterol to high-density lipoprotein cholesterol ratio; aHR, adjusted hazard ratio; CI, confidence interval; MACE, major adverse cardiovascular events. | | | | | |
